# Impact of gonadectomy on blood pressure regulation in ageing male and female rats

**DOI:** 10.1186/s13293-016-0111-9

**Published:** 2016-12-03

**Authors:** Wioletta Pijacka, Bethan Clifford, Dawid Walas, Chantal Tilburgs, Jaap A. Joles, Sarah McMullen, Simon C. Langley-Evans

**Affiliations:** 1 Division of Nutritional Sciences, School of Biosciences, University of Nottingham, Sutton Bonington Campus, Loughborough, UK; 2 School of Physiology, Pharmacology and Neuroscience, University of Bristol, University Walk, Bristol, BS8 1TD UK; 3 Department of Nephrology & Hypertension, University Medical Centre, Utrecht, The Netherlands

**Keywords:** Gonadectomy, Blood pressure, Renal function

## Abstract

**Background:**

Sexual dimorphism in blood pressure has been associated with differential expression of the angiotensin II (AII) receptors and with activity of the nervous system. It is generally accepted that ageing affects kidney function as well as autonomic nervous system and hormonal balance. Given that hypertension is more prevalent in men than women until women reach their seventh decade, we hypothesised that females would be relatively protected from adverse effects of ageing compared to males and that this would be mediated by the protective effect of ovarian steroids.

**Methods:**

Intact and gonadectomised male and female normotensive Wistar rats aged 6, 12 and 18 months were used to study renal function, blood pressure, heart rate, and blood pressure variability.

**Results:**

We observed that intact females had lower levels of proteinuria and higher (12.5%) creatinine clearance compared to intact males and that this difference was abolished by castration but not by ovariectomy. Ovariectomy resulted in a change by 9% in heart rate, resulting in similar cardiovascular parameters to those observed in males or gonadectomised males. Spectral analysis of systolic blood pressure revealed that high-frequency power spectra were significantly elevated in the females vs. males and were reduced by ovariectomy.

**Conclusions:**

Taken altogether, the results show that females are protected from age-related declining renal function and to a lesser extent from rising blood pressure in comparison to males. Whilst ovariectomy had some deleterious effects in females, the strongest effects were associated with gonadectomy in males, suggesting a damaging effect of male hormones.

**Electronic supplementary material:**

The online version of this article (doi:10.1186/s13293-016-0111-9) contains supplementary material, which is available to authorized users.

## Summary statement

Better understanding on how age affects interaction between sex hormones, AngII and autonomic nervous system could help choosing an optimal treatment for hypertensive patients.

## Background

The level of blood pressure is proportionally related to morbidity and mortality, and increases in blood pressure convey a risk of stroke and coronary heart disease [1, 2]. This risk is further elevated when associated with other conditions such as obesity or diabetes. Sexual dimorphism in blood pressure regulation has been extensively studied and both systolic (SBP) and diastolic blood pressures (DBP) are higher in men under 60 years of age compared to age-matched women [3, 4]. The incidence of hypertension is greater in men than women until women reach their seventh decade [5, 6]. This increased risk of hypertension in men is associated with a higher risk of developing renal and cardiovascular diseases [7]. Interestingly, blood pressure increases in women after the menopause leading to speculation that the protective effect of female gender may be mediated by oestrogen, with testosterone exerting opposing effects [8–11].

Sexual dimorphism in blood pressure has been associated with differential expression of the angiotensin II (AII) receptors [12–15] and correlated with activity of the nervous system. In general, AII increases blood pressure on binding to angiotensin receptor type 1 (AGTR1) and decreases blood pressure when acting via angiotensin receptor type 2 (AGTR2) [16]. AGTR1 expression has been shown to be related to the presence or absence of oestrogen but not testosterone in normotensive and hypertensive male rats [12, 17]. Renal AGTR2 expression was shown to be higher in female spontaneously hypertensive rats (SHR) compared to male SHR [17, 18] and binding of AGTR2 was reduced after ovariectomy but returned to normal or even increased after oestrogen treatment. These findings suggest that AGTR2 and AGTR1 expression may be responsive to sex steroids and that the relative protection of the female against age-related increases in blood pressure could stem from higher AGTR2 expression. Sexual dimorphism in renal AII receptor expression is also associated with differences in renal hemodynamics [19, 20]. It has been shown that AGTR1 regulates vasoconstriction and sodium and water reabsorption, as well as promotes cell growth, proliferation, and collagen matrix deposition. AGTR2 seems to exert opposite effects such as promoting cell differentiation, antiproliferation, and apoptosis; for review, see [21].

The complex interactions between age and sex steroids in the regulation of blood pressure remain poorly understood. The majority of studies investigating the impact of sex steroids focus on single age points or relatively short follow-up periods. The current study investigated the interactions between ageing and sex steroids in the normotensive Wistar-Hanover rat, comparing intact and gonadectomised animals up to 18 months of age. The purpose of the study was to model the impact of sex on functional aspects of cardiovascular and renal ageing. As sex-related factors will act upon tissues throughout adult life, we performed ovariectomy in mature but relatively young animals. This study assessed the hypothesis that females would be relatively protected from adverse effects of ageing compared to males and that this would be mediated either by protective effects of ovarian steroids in females or detrimental effects of testosterone in males.

## Methods

### Animals

All animal procedures were performed in accordance with the UK Animals (Scientific Procedures) Act of 1986. Rats (Wistar HsdHan), bred in the University of Nottingham animal facility, (64 males and 64 females) were exposed to 12-h day-night cycle. At 10 weeks of age, all animals underwent gonadectomy or sham gonadectomy. Females underwent an ovariectomy or sham surgery and males castration or sham surgery under isoflurane anaesthesia in sterile conditions. The protocol for ovariectomy of females has been described [20]. Castration consisted of opening the scrotal sac, applying suture on each main visible artery and taking out both testicles whereas during sham surgery, the scrotal sac was opened and then immediately closed. The surgical protocols hence generated four treatment groups: intact females (F-INTACT), ovariectomised females (F-GONADX), intact males (M-INTACT) and castrated males (M-GONADX). Rats of each gender were randomly assigned to GONADX or INTACT. Prior to sacrifice, the animals were placed in metabolic cages for 24-h urine collection. Whilst in metabolic cages, the rats had unlimited access to food and water. The animals were then killed by carbon dioxide asphyxia and cervical dislocation at 6, 12 or 18 months of age (*n* = 8 per group at each time point). Following euthanasia, all tissues were weighed and snap-frozen in liquid nitrogen and then stored at −80 **°**C until processing or immediately fixed in buffered formaldehyde and embedded after a constant fixation period. The tissues were analysed at the same time for each parameter under study. Blood was collected by cardiac puncture into lithium-heparin micro-tubes (Sarstedt, Leicester, UK).

At 12 months of age, eight males and eight females per group were implanted with telemetry transmitters in the descending aorta (PA-C40; DSI, USA). All surgeries were performed in sterile conditions under isofluorane anaesthesia [22]. All rats were given a semi-synthetic opioid (0.0168 ml/100 g buprenorphine; 0.3 mg/ml, Reckitt & Colman, Slough, UK) and a non-steroidal anti-inflammatory drug (0.004 ml/100 g Metacam; 1.5 mg/ml, Boehringer Ingelheim, Germany) post-surgery as pain relief. The rats were single housed after surgery, and they were allowed 1 week of recovery prior to commencing blood pressure measurements.

### Blood pressure measurements

At 12 months of age, the rats had their blood pressure determined initially by telemetry and later using an indirect tail-cuff method (CODA, Kent Scientific, USA). Telemetry recordings were performed on a DSI Dataquest A.R.T.™ acquisition system and arterial blood pressure was continuously recorded over four consecutive days at 1 kHz [20, 22]. During this time, the rats were singly housed with free access to food and water during the data collection. Indirect tail-cuff blood pressure measurements were taken between 10 am and 2 pm on the day following the day of the last telemetry recording. Tail-cuff measurements were performed again at 18 months of age on the older group of females and males. Telemetry was not performed at this time point as there were concerns about animal welfare and recovery of older animals from major surgery.

### Telemetry analysis

Arterial pressure was sampled at 1 kHz using DSI Dataquest A.R.T.™ acquisition system. From the arterial pressure, we derived pulse pressure and heart rate (HR). Blood pressure and heart rate were collected over four consecutive days, then data were averaged per active and resting phase across the measurement period. Data are presented as an average day. In order to analyse respiratory rate and power spectra of heart rate and blood pressure, the data were imported into Spike 2 v8.02c 64-bit via CED’s custom batch import script. Additionally, the spontaneous cardiac baroreflex gain was also analysed by Scripts (second edition August 2013).

### Biochemical assays

Renal function was assessed through the measurement of urinary protein, albumin, plasma urea, and creatinine clearance. As described [23], plasma and urinary creatinine were measured by the improved Jaffe method (Universal Biologicals, Cambridge, UK). Urinary albumin was measured by the improved BCG method (Randox Laboratories Ltd., Crumlin, UK). Total urinary protein was measured by the Lowry method with the DC Protein Assay Kit (Bio-Rad Laboratories Ltd, Hemel Hempstead, UK) [24]. Plasma urea was measured by the Jung method (urea assay kit; Universal Biologicals).

### Western blotting

The kidney cortex was crushed in liquid nitrogen with a mortar and pestle and then homogenised with protein extraction buffer as described previously [20]. Protein concentration was established using the DC Protein Assay Kit. Expression of AGTR1 and AGTR2 proteins was then measured using the Amersham ECL Plex Western blotting system using low-fluorescent PVDF membrane (GE Healthcare, Buckinghamshire, UK) as described previously [20]. Briefly, anti-AGTR1 ab18801 and anti-AGRT2 ab19134 antibodies were used. We have reported the optimisation of anti-AGRT2 ab19134 [20]. Anti-histone H2B (ab52484) primary antibody at 1:40000 dilution incubation with membrane for 1 h at room temperature was followed by washing and then incubation with secondary antibody as described previously [20]. Signal on the membrane was detected by fluorescent laser scanner (Typhoon, GE Healthcare, Buckinghamshire, UK), and images were quantified using ImageQuant (GE Healthcare). The optimal primary and secondary antibody concentrations were tested. Specificity of AGTR1 (Additional file 1: Figure S1) and AGTR2 [20] antibodies was confirmed by blocking peptide: AGTR1 -ab91523 (Abcam, Cambridge, UK) and AGTR2 -ab91522 (Abcam). Expression of AGTR1 and AGTR2 was normalised to histone H2B expression, which did not vary between males and females, with age or surgical treatment.

### Histology

The kidneys were fixed with 4% formalin (Sigma-Aldrich, UK) immediately after collection. The fixed kidney samples were processed and embedded in paraffin using a tissue processor (Histokinette Benchtop, Fullerton, CA, USA). To assess tissue inflammation, macrophages were stained with an antibody to ED1 (Abcam, Ab31630) [25]. Lymphocytes were stained with an antibody to CD3 (RbαHumanCD3: DAKO A0452) [26]. Bright vision-HRP was used as secondary antibody (Immunologic, Tilburg, Netherlands). Positive cells were visualised with Vector Nova Red (Vector) and counterstained with haematoxylin. ED1- and CD3-positive cells were counted in the left kidney. In the kidney sections, positive cells in 50 glomeruli and 20 peritubular areas were counted (magnification ×400) [20].

### Statistical analysis

All data are presented as mean ± SEM for eight observations per group unless stated otherwise. Data was analysed by three-way ANOVA (SPSS, Chicago, IL, USA) with age and sex steroids and gonadectomy as fixed factors, followed by Tukey post hoc analysis. Eta squared has been reported for the effect size when appropriate.

## Results

### General characteristics and renal function

The general characteristics of the animals (body weights, left kidney and heart weights) are shown in Table 1. As expected, males had significantly higher body weight compared to females, *P* < 0.001. M-GONADX had lower body weight than M-INTACT, whereas F-GONADX was heavier than F-INTACT (*P* < 0.001). Overall body mass increased with age by 42% in all groups (*P* < 0.001; *η* ^2^ = 0.24). Heart weight was smaller at 12 months vs. 6 and 18 months. The hearts were smaller in the females vs. males (*η* ^2^ = 0.28), and GONADX had decreased heart weight in both sexes, *P* < 0.001; *η* ^2^ = 0.16. Left kidney weights were lower in older animals (*η* ^2^ = 0.06), and females had smaller kidneys than males (*P* < 0.001; *η* ^2^ = 0.46). GONADX had decreased kidney weights in both sexes, (*P* < 0.001; *η* ^2^ = 0.42).

**Table 1:** General characteristics of male and female rats at 6, 12, and 18 months of age

General characteristics	6 months				12 months				18 months				Three-way ANOVA
	F-INTACT	F-GONADX	M-INTACT	M-GONADX	F-INTACT	F-GONADX	M-INTACT	M-GONADX	F-INTACT	F-GONADX	M-INTACT	M-GONADX	
BW (g)	264.7 ± 18.1	287.8 ± 19.4	425.1 ± 11.8	398 ± 18.1	295.9 ± 20.9	350.6 ± 20.9	504.3 ± 18.3	427.1 ± 18.1	372.4 ± 18.1	422.1 ± 18.1	627.1 ± 24.3	533.4 ± 18.1	6 < 12 < 18*** F < M***; Sex*GONADX***
Kidney left (g)	0.8 ± 0.05	0.9 ± 0.05	1.4 ± 0.05	1.0 ± 0.05	1.1 ± 0.07	0.9 ± 0.07	1.5 ± 0.05	1.1 ± 0.05	1.0 ± 0.05	1.0 ± 0.05	1.5 ± 0.05	1.1 ± 0.05	6 < 12,18***; F < M***; Sham > GONADX***; Gender*GONADX***
Heart (g)	0.9 ± 0.06	0.9 ± 0.06	1.5 ± 0.06	1.1 ± 0.06	1.2 ± 0.06	0.9 ± 0.07	1.2 ± 0.06	1.0 ± 0.06	1.1 ± 0.06	1.0 ± 0.06	1.5 ± 0.06	1.2 ± 0.06	6 > 12 < 18***; F < M***; Sham > GONADX***; Gender*GONADX***
Urine volume (ml/24 h)	11.0 ± 1	9.9 ± 1	11.1 ± 1	9.7 ± 1	13.4 ± 1	10.1 ± 2	10.1 ± 1	10. ± 1	10.9 ± 1	7.9 ± 1	11.3 ± 1	15.2 ± 1	Sex*GONADX*, Sex*Age*
Water intake (ml/24 h)	27.5 ± 2	18.3 ± 2	22.8 ± 2	20.6 ± 2	26.2 ± 3	20.9 ± 3	23.6 ± 2	21.1 ± 2	24.9 ± 2	18.9 ± 2	22.1 ± 2	24.4 ± 2	Sham > GONADX***, Sex*GONADX*

Animals underwent sham (INTACT) or ovariectomy or castration (GONADX) surgery at 10 weeks of postnatal age. The three-way ANOVA assessed the main factor effects of age, sex steroid and surgery, with Tukey HSD post hoc analysis where applicable. Data are presented as mean ± SEM for *n* = 8 animal per group

*FI* female INTACT, *FG* female GONADX, *MI* male INTACT, *MG* male GONADX,

*P < 0.05; ***P < 0.001

**Fig. 1:**
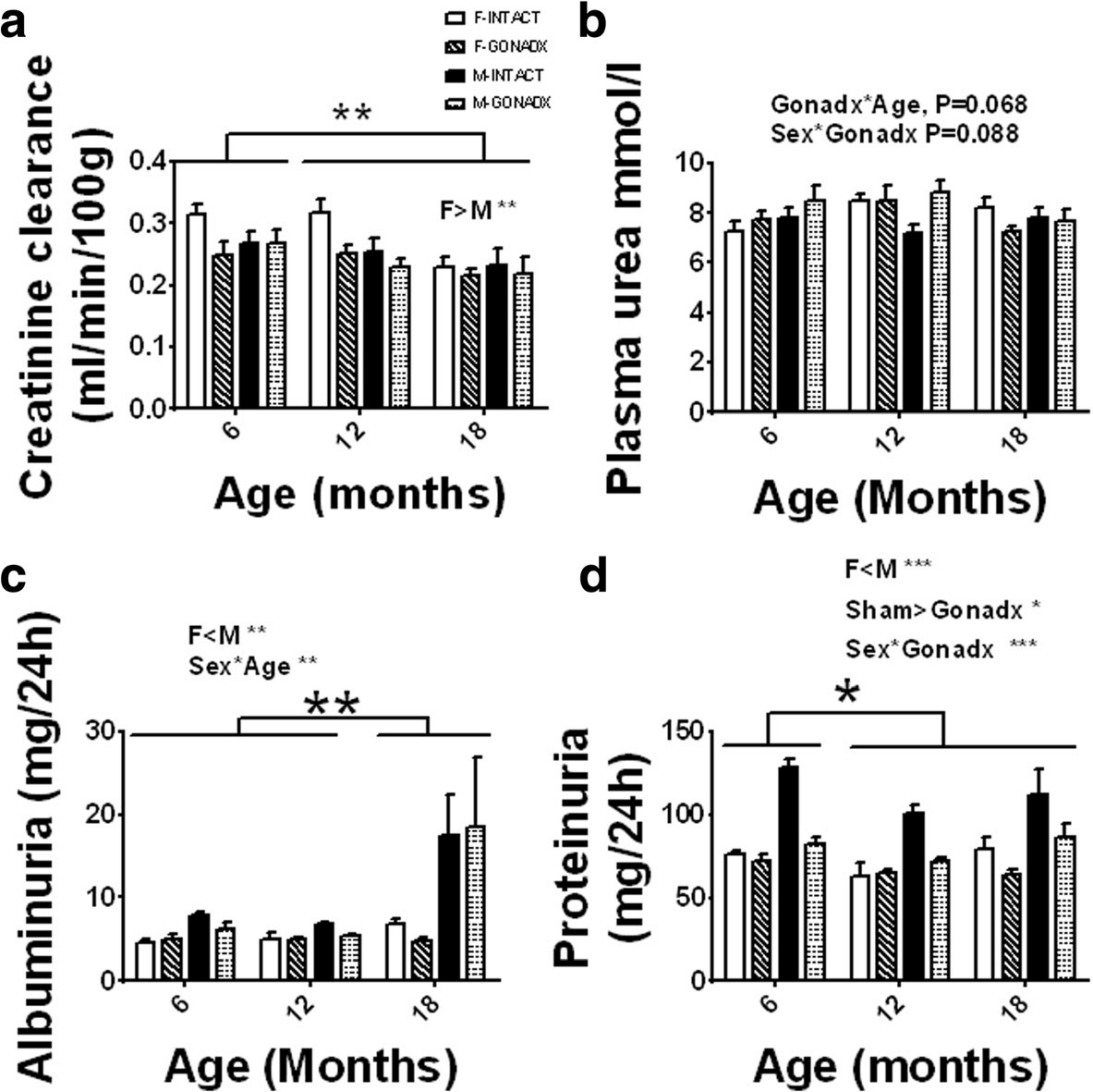
Kidney function. a﻿ Glomerular filtration rate (GFR) based on the creatinine clearance over a 24-h period and adjusted for the body weight of the animal showed decline in renal function with ageing (6, 12 > 18 months of age, *P* < 0.01). Females had higher GFR than males (*P* < 0.05). b The plasma urea concentration was unaffected by either ageing or surgery although there was a tendency towards change in the concentration of urea with age after gonadectomy *P* = 0.068. c Albuminuria was higher in males vs. females (*P* < 0.01) and the amount of albumin excreted increased with age (6, 12 < 18 months of age, *P* = 0.01), though this effect was largely confined to males. d Proteinuria was higher in males vs. females (*P* < 0.001). Proteinuria also increased with age in males (6 < 12, 18 months of age, *P* < 0.05). The INTACT animals had higher proteinuria than the GONADX, and this was significantly reduced by castration (*P* < 0.001). Data are presented as mean ± SEM for *n* = 8 per group,*P < 0.05; **P < 0.01; ***P < 0.001

Glomerular filtration rate (GFR), based on the creatinine clearance over a 24-h period (Fig. 1) and adjusted for the body weight of the animal, showed decline in renal function with ageing which was significant at 18 months of age (6, 12 > 18 months of age, *P* < 0.01; *η* ^2^ = 0.02). Overall females had higher GFR than males by 12.5% (*P* < 0.05; *η* ^2^ = 0.29). Water intake and the urine production were influenced by sex and GONADX. The INTACT animals consumed much more fluid (by 18%) over the 24-h period than the GONADX rats (*P* < 0.001; *η* ^2^ = 0.10). Water intake was unaffected by GONADX in males. There was an interaction between age and sex in terms of urine output (*P* < 0.05). This was associated with a progressive decline (by 18%) in urine output in F-GONADX but not F-INTACT with ageing, *η* ^2^ = 0.06 (Table 1).

The plasma urea concentration was unaffected by either ageing or surgery although there was a tendency towards change in the concentration of urea with age after gonadectomy *P* = 0.068 (Fig. 1). Albuminuria was higher in males vs. females by 50% (*P* < 0.01; *η* ^2^ = 0.09) and the amount of albumin excreted increased with age (6, 12 < 18 months of age, *P* = 0.01; *η* ^2^ = 0.12), though this effect was largely confined to males (61% change between 18-month-old females and males). Proteinuria was higher in males vs. females by 36% (*P* < 0.001; *η* ^2^ = 0.27). Proteinuria also increased with age in males, (6 < 12, 18 months of age, *P* < 0.05; *η* ^2^ = 0.04). The INTACT animals had higher proteinuria than GONADX, and this was significantly reduced by castration (*P* < 0.001; *η* ^2^ = 0.16).

### Inflammatory response in kidney cortex

**Fig. 2:**
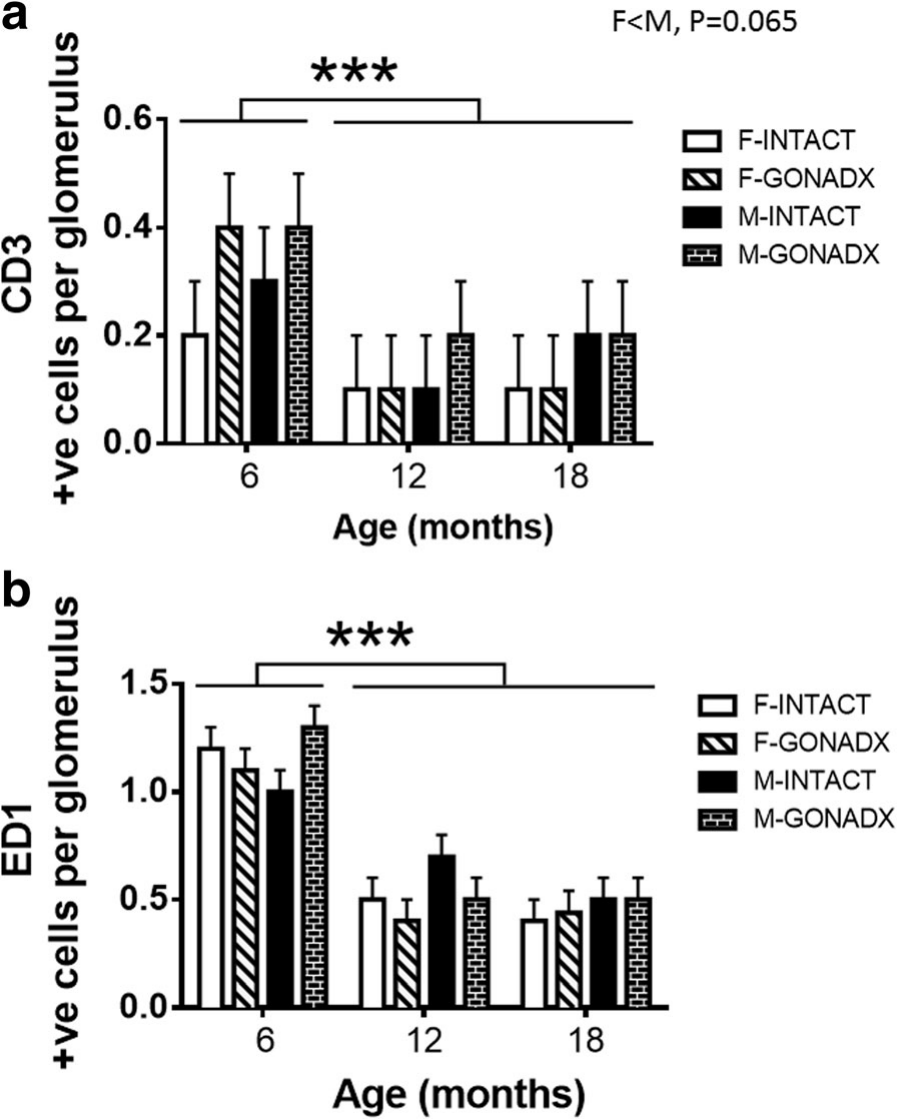
Inflammatory markers. Immunohistochemistry on the left sectioned kidney shows CD3 (**a**) and ED1 (**b**) inflammatory markers expressed as positive cells per glomerulus of the kidney. CD3, a T cell marker, was increased in the glomeruli at 6 months compared to that of 12 and 18 months of age, *P* < 0.01. ED1, a monocyte and macrophage marker, was increased in the glomeruli at 6 months compared to that of 12 and 18 months of age, *P* < 0.01. There were no influences of gonadectomy. Data are presented as mean ± SEM for *n* = 8 per group, ***P < 0.001

**Fig. 3:**
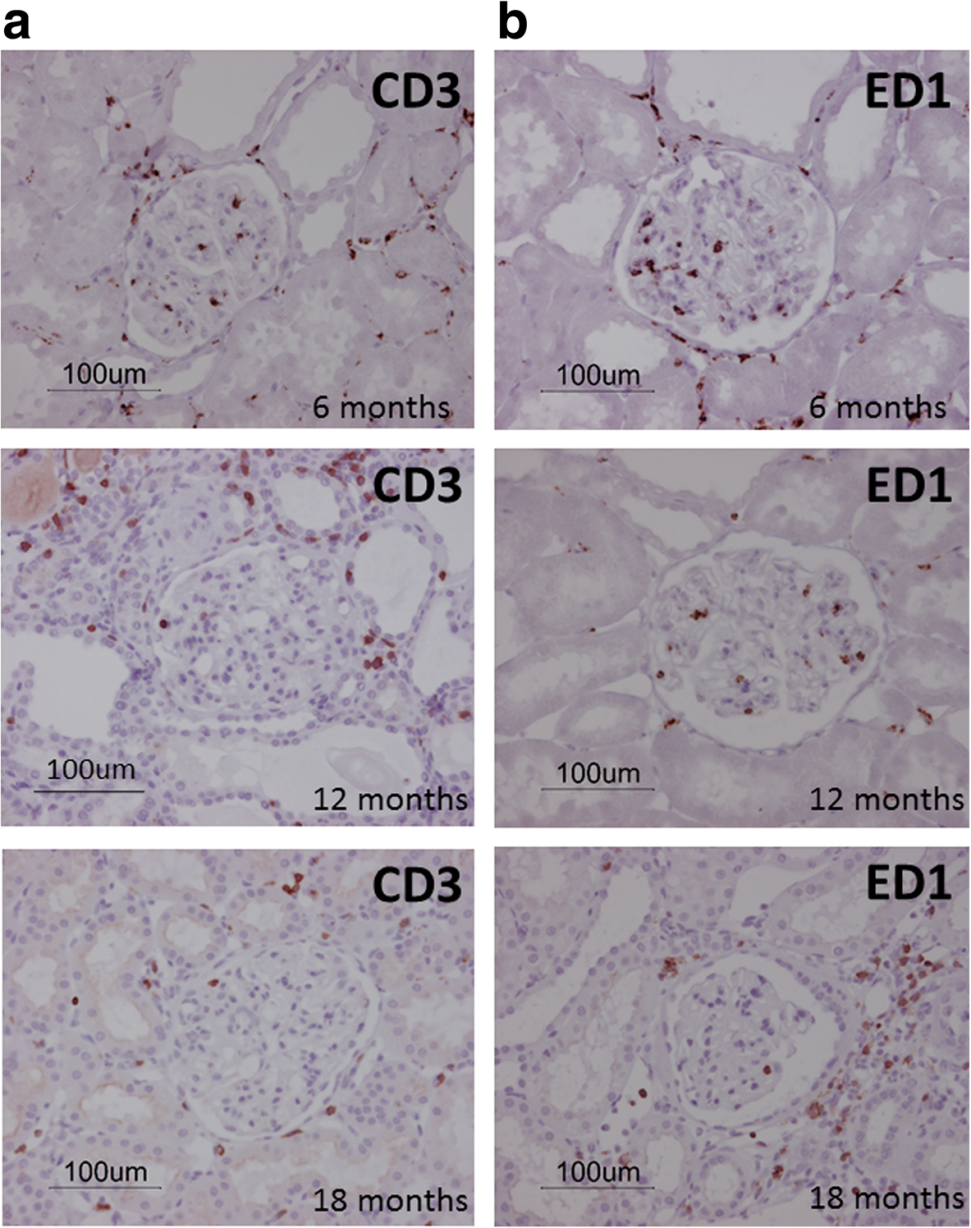
Representative images of inflammatory markers. Representative images of CD3 (**a**) and ED1 (**b**) in glomerulus of the left sectioned kidney at 6, 12 and 18 months of age

CD3, a T cell marker, was more strongly expressed in the glomeruli at 6 months of age compared to 12 and 18 months of age (*P* < 0.001; *η* ^2^ = 0.19, Figs. 2a and 3a). Partial correlation showed that there is no correlation between T cell numbers in glomerulus and kidney size (g) during ageing. There was a trend for females to have less CD3 than males (*P* = 0.065). Staining for ED1, a monocyte and macrophage marker, was greater in the glomeruli at 6 months of age compared to 12 and 18 months of age (*P* < 0.01; *η* ^2^ = 0.50, Figs. 2b and 3b). There were no effects of GONADX on T cell and macrophage markers in the kidneys. There were no effects of age or surgery on inflammatory cell markers in the tubulointerstitial region (data not shown). There were no marked changes in conventional histology (PAS stain, data not shown). Higher levels of proteinuria in male rats are well known and primarily due to tubular proteinuria [27, 28].

### Angiotensin receptor expression in ageing females and males

**Fig. 4:**
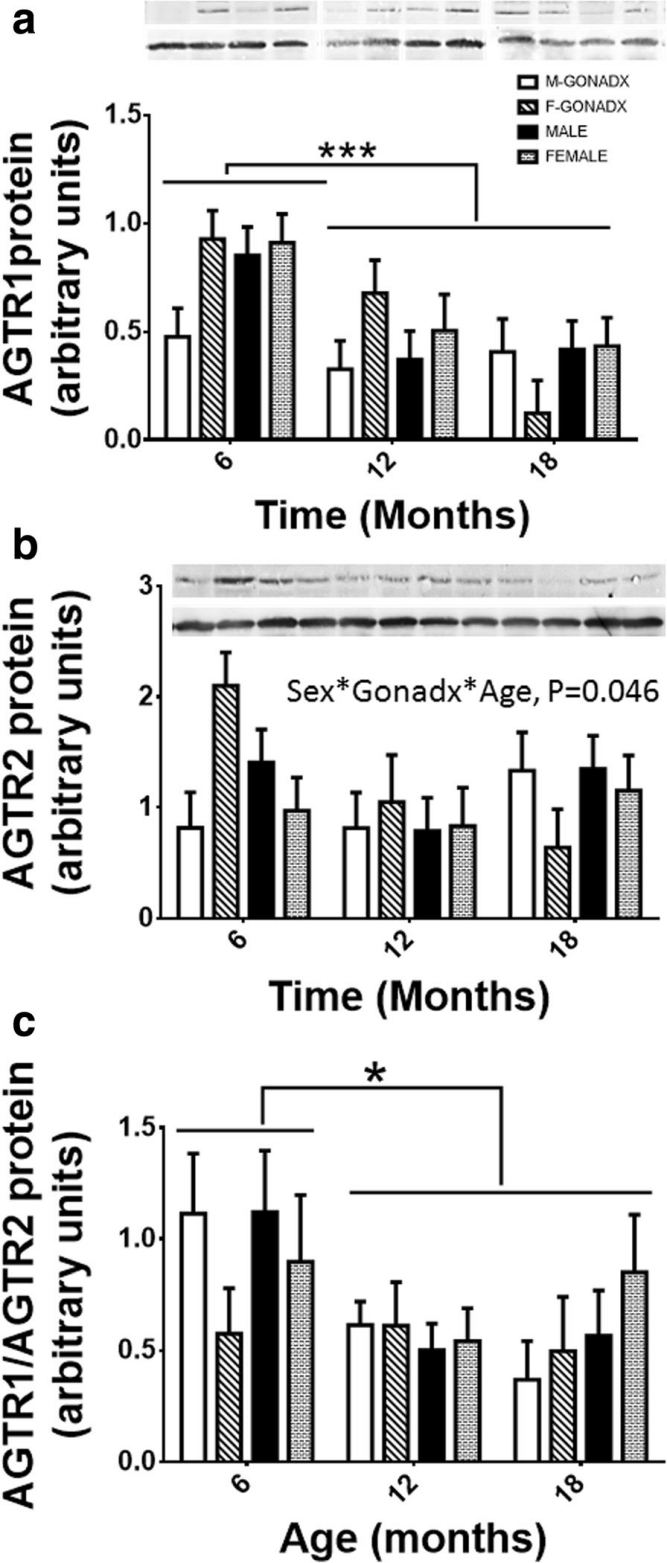
AGTR1 and AGTR2 protein expression. Kidney cortex protein level of AGTR1 (**a**) and AGTR2 (**b**) was assessed by Western blotting, and the AT1/AT2 (**c**) ratio presents a change of these receptors at 6, 12 and 18 months of age in relation to surgery. The AGTR1/AGTR2 ratio was significantly increased at 6 months of age, *P* < 0.05. Data are presented as mean ± SEM for *n* = 8 animal per group, *P < 0.05; ***P < 0.001

AGTR1 expression in the kidney cortex decreased significantly with age in all analysed groups (6 > 12, 18 month of age, *P* < 0.001; *η* ^2^ = 0.20, Fig. 4a). We did not see a significant difference in AGTR1 protein expression between males and females. AGTR2 protein expression in the kidney cortex decreased with age after GONADX in females by 46% (*P* < 0.05; *η* ^2^ = 0.02, Fig. 4b). There was a decrease in the AGTR1/AGTR2 ratio with age (6 > 12, 18 months of age, *P* < 0.05; *η* ^2^ = 0.09, Fig. 4c).

### Blood pressure and heart rate

**Fig. 5:**
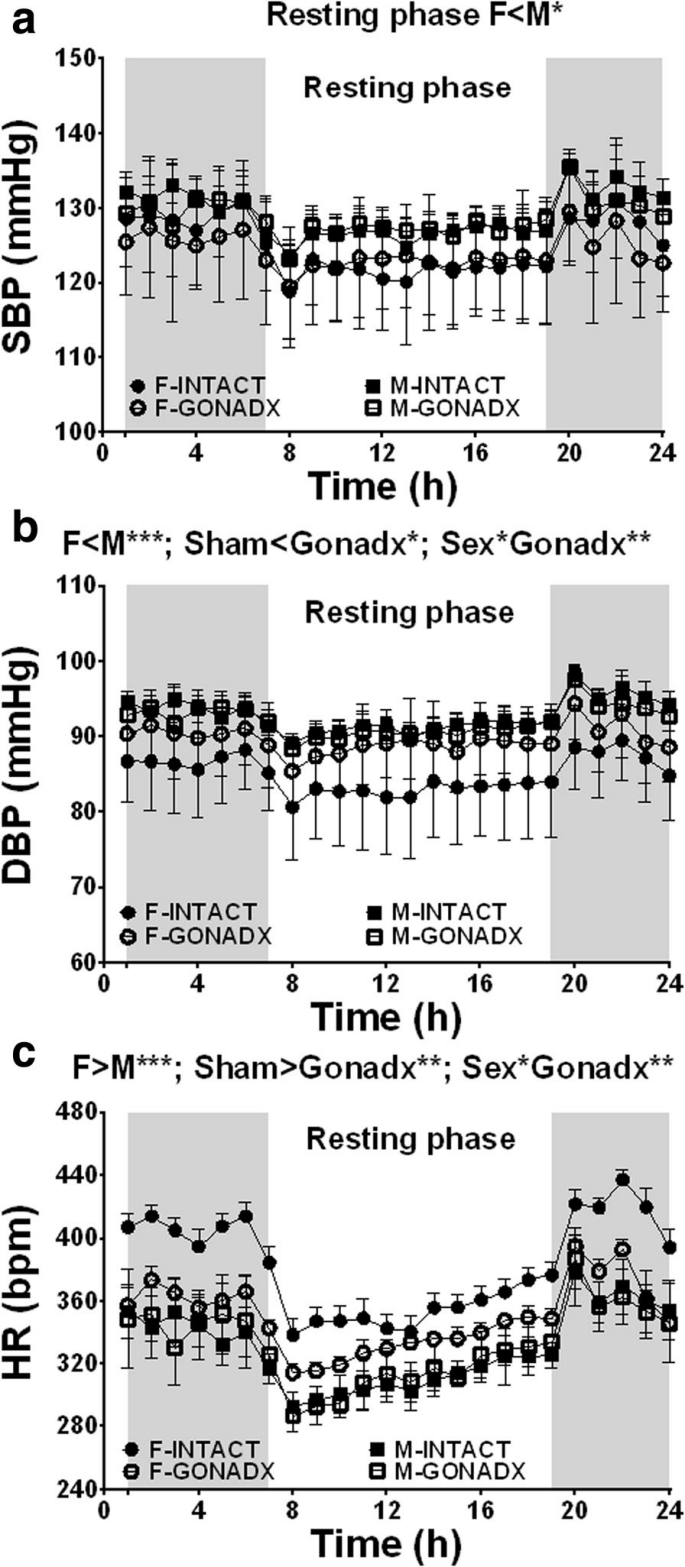
Mean arterial blood pressure (mmHg) and heart rate (bpm) assessed telemetrically. Arterial blood pressure and heart rate were assessed telemetrically at 12 months of age. Females had a lower systolic blood pressure than males (SBP (**a**)), *P* < 0.05. The diastolic blood pressure (DBP (**b**)) was lower in females (*P* < 0.001) and in sham animals (*P* < 0.05), and there was an effect of ovariectomy (*P* < 0.01). Females mean heart rate (HR (**c**)) was significantly higher than all other groups (*P* < 0.001), and this was reduced by ovariectomy (*P* < 0.01). Additionally, sham animals had higher HR than gonadectomised rats (*P* < 0.05). Data are presented as mean ± SEM for *n* = 8 animal per group,*P < 0.05; **P < 0.01; ***P < 0.001

Telemetry data analysis show that at 12 months of animal age, females had significantly lower SBP compared to males by 4% (*P* < 0.05; *η* ^2^ = 0.18, Fig. 5a). DBP was also lower in females as compared to males, by 8% (*P* < 0.001; *η* ^2^ = 0.25) and lower in INTACT animals when compared to GONADX animals (*P* < 0.05; *η* ^2^ = 0.47). We observed that there was also an interaction between gender and GONADX showing that ovariectomy increased SBP (Fig. 5b, *P* < 0.01). Mean heart rate was significantly higher in females than in males by 14%, and the INTACT animals had higher HR than the GONADX rats (Fig. 5c, *P* < 0.001; *η* ^2^ = 0.51). Additionally, GONADX reduced the HR in females (Fig. 5c, *P* < 0.05; *η* ^2^ = 0.64). Respiratory rate was greater in female than in male rats, and this was not influenced by GONADX (*P* < 0.001; *η* ^2^ = 0.44 Table 2).

**Table 2:** Spectral power analysis of the heart rate (HR) in normotensive intact and gonadectomised rats

Variable	F-INTACT (*n* = 7)	F-GONADX (*n* = 7)	M-INTACT (*n* = 8)	M-GONADX (*n* = 5)	Two-way ANOVA
TP bpm^2/^Hz	4.0 ± 1.3	6.3 ± 1.2	4.6 ± 1.1	3.3 ± 1.4	NS
VLF bpm^2/^Hz (0–0.26 Hz)	2.8 ± 0.5	2.8 ± 0.5	2.3 ± 0.4	1.7 ± 0.6	NS
LF bpm^2/^Hz (0.26–0.76 Hz)	0.5 ± 0.2	1.0 ± 0.2	0.6 ± 0.2	0.5 ± 0.2	NS
HF bpm^2/^Hz (0.76–3.3 Hz)	0.7 ± 0.1	2.5 ± 1.1	1.8 ± 0.7	1.1 ± 0.4	NS
LF:HF	0.7 ± 0.1	0.6 ± 0.1	0.5 ± 0.1	0.6 ± 0.1	NS
VLF % (HR)	68.1 ± 7.6	55.0 ± 7.1	53.3 ± 6.6	53.4 ± 8.4	NS
LF % (HR)	12.0 ± 2.2	15.0 ± 2.0	12.6 ± 1.9	15.4 ± 2.4	NS
HF % (HR)	11.6 ± 6.8	29.9 ± 6.3	34.6 ± 5.9	31.2 ± 7.5	NS

Data analysed by Spike2 v 8.02c (CED, Cambridge, UK). The two-way ANOVA assessed the effect of sex steroids and surgery, with Tukey HSD post hoc analysis. Data are presented as mean ± SEM

*TP* total spectrum power, *VLF* very low frequency spectrum, *LF* low-frequency spectrum, *HF* high-frequency spectrum, *NS* non-significant

**Fig. 6:**
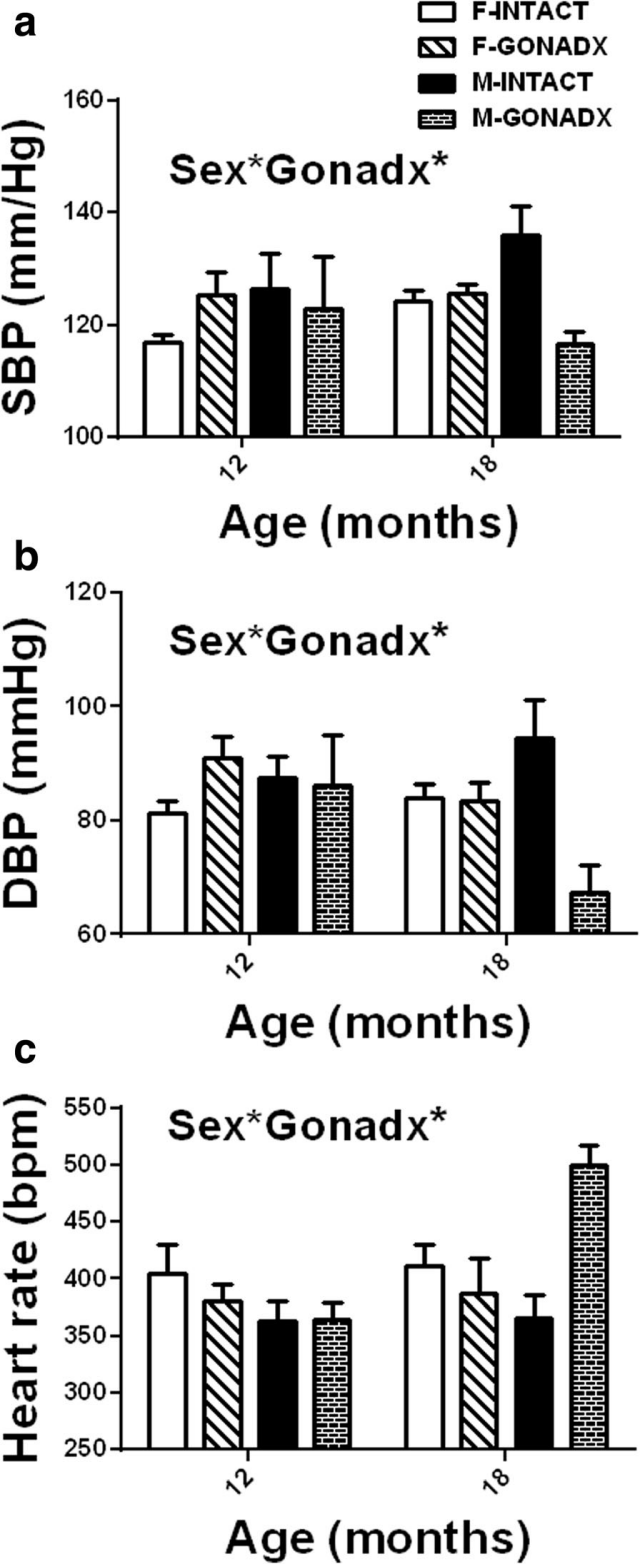
Mean arterial blood pressure (mmHg) and heart rate (bpm) measured by tail-cuff system CODA. The SBP (**a**) and DBP (**b**) was decreased by castration that was more pronounced at 18 months of age (interaction age × surgery, *P* < 0.05). There was a significant increase in the heart rate (HR; **c**) in the M-GONADX at 18 months of age, *P* < 0.05. Data are presented as mean ± SEM for *n* = 8 animal per group,*P < 0.05

Blood pressure was also recorded by an indirect tail-cuff method. Using plethysmography, we noted that the SBP recorded at 12 and 18 months showed a tendency to be higher in males than in females (Fig. 6a, *P* = 0.07). Additionally, a comparison of SBP between 12 and 18 months in females (*T* test) showed a significant difference by 6% (*P* < 0.01). In contrast to telemetry measures, DBP was lower in GONADX males than in INTACT males at 12 months and this effect was more pronounced at 18 months, 40% lower (Fig. 6b, interaction age × surgery, *P* = 0.064). HR increased with age (Fig. 6c, *P* < 0.05; *η* ^2^ = 0.07). At 18 months, GONADX males had markedly higher heart rates than all other groups (*P* < 0.05).

### Spectral analysis of heart rate and systolic blood pressure

Spectral analysis performed on the heart rate data obtained by telemetry at 12 months of age showed no effects of either age or GONADX (Table 2). The lack of differences in low-frequency and high-frequency spectra indicated that sympathetic and parasympathetic regulation of heart rate were unaffected by sex steroids at this time point. In contrast, spectral analysis of SBP revealed that low as well as high-frequency power spectra changed in males and females upon GONADX. Females had a higher HF% which was reduced by GONADX (Table 3, *P* < 0.05; *η* ^2^ = 0.48). Females show lower VLF% compared to males (Table 3, *P* < 0.05). The spontaneous baroreflex gain was higher in males than in females (*P* < 0.05; *η* ^2^ = 0.17, Table 3).

**Table 3:** Spectral power analysis of the SBP and sBRG in normotensive intact and gonadectomised rats

Variable	F-INTACT (*n* = 7)	F-GONADX (*n* = 7)	M-INTACT (*n* = 8)	M-GONADX (*n* = 5)	Two-way ANOVA
TP mmHg^2/^Hz	0.24 ± 0.03	0.27 ± 0.03	0.21 ± 0.03	0.32 ± 0.03	Sham < GONADX*
VLF mmHg^2/^Hz (0–0.26 Hz)	0.06 ± 0.02	0.09 ± 0.02	0.09 ± 0.02	0.11 ± 0.02	NS
LF mmHg^2/^Hz (0.26–0.76 Hz)	0.09 ± 0.02	0.12 ± 0.02	0.06 ± 0.02	0.12 ± 0.02	Sham < GONADX*
HF mmHg^2/^Hz (0.76–3.3 Hz)	0.08 ± 0.01	0.07 ± 0.01	0.05 ± 0.01	0.08 ± 0.01	Sex*GONADX**
LF:HF	1.1 ± 0.2	1.7 ± 0.2	1.6 ± 0.2	1.5 ± 0.2	Sex*GONADX P = 0.08
VLF % (SBP)	25.7 ± 5.3	30.6 ± 5.3	43.3 ± 4.4	35.8 ± 5.3	F < M*
LF % (SBP)	36.8 ± 4.0	42.5 ± 4.0	34.3 ± 3.4	37.9 ± 4.0	NS
HF % (SBP)	37.5 ± 3.4	26.9 ± 3.4	22.4 ± 2.8	26.3 ± 3.4	F > M*; Sex*GONADX*
Respiratory rate 24 h (breath per min)	96.3 ± 3.7	91.8 ± 4.0	76.5 ± 3.2	78.1 ± 4.0	F > M***
sBRG (+)	1.1 ± 0.1	1.2 ± 0.1	1.3 ± 0.1	1.5 ± 0.1	F < M*
sBRG (−)	−1.5 ± 0.2	−1.8 ± 0.1	−1.9 ± 0.2	−2.3 ± 0.2	F < M*

Data analysed by Spike2 v 8.02c (CED, Cambridge, UK). The two-way ANOVA assessed the effect of sex steroids and surgery, with Tukey HSD post hoc analysis. Data are presented as mean ± SEM

*TP* total spectrum power, *VLF* very low frequency spectrum, *LF* low-frequency spectrum, *HF* high-frequency spectrum, *SBP* systolic blood pressure, *sBRG (+)* positive ramp of spontaneous baroreflex gain, *sBRG (−)* negative ramp of spontaneous baroreflex gain, *NS* non-significant

*P < 0.05; **P < 0.01;***P < 0.001

## Discussion

The present study investigated mechanisms contributing to sexual dimorphism in blood pressure in ageing rats. It is generally accepted that ageing affects kidney function as well as the autonomic nervous system and hormonal balance. Our original hypothesis was that the relative protection of renal and cardiovascular function in ageing females was due to the presence of ovarian steroids (primarily oestrogen) or detrimental effects of testosterone in males. Additionally, age-related changes in the autonomic balance of the sympathetic/parasympathetic nervous system might contribute to sex differences. The novel aspect of this work is that we gonadectomised animals in early adulthood and followed them as they aged. We looked at the renal function of intact and gonadectomised normotensive Wistar rats of both sexes in relation to blood pressure changes over a period of 18 months. The main findings of this study confirmed the view that females are protected against deteriorating kidney function and have lower arterial pressure than males. This appears to be largely explained by deleterious effects of testosterone and other androgens upon males, rather than protective effects of ovarian steroids in females. Androgens may partly exert their influence via the autonomic nervous system.

Sex steroids are responsible for the development of secondary characteristics in almost every species [29]. As expected, all rats in our study showed an increase in body mass across the life span, and there were clear differences between sexes and gonadectomised rats. Ablation of sex steroid synthesis by gonadectomy increased weight gain in females and reduced gain in males, consistent with other studies [30, 31]. Organ weights revealed that the absence of sex steroids had an impact on kidney and heart development. These organs were lighter in older animals, and they were smaller in gonadectomised animals (Table 1). Sex steroids appear to affect kidney development and function. Kidney size is postulated to be related to the number of glomeruli, and putatively, a decreased number of functioning nephrons in the male kidney may cause glomerular hypertension. The effect of testosterone on the number of the glomeruli was recently published [32]. Testosterone replacement therapy to castrated rats resulted in a higher kidney-to-body weight ratio but with reduced numbers of glomeruli. The number of glomeruli was highest in the castrated group indicating that testosterone promotes a progressive loss of glomeruli with ageing in males. In the present study, creatinine clearance, an estimation of glomerular filtration rate, indicated that kidney function declined with age but that females were relatively protected. Creatinine clearance was lower in males than in females which may suggest that ovarian steroids partly protect against age-related decline.

Under our conditions, we observed that females had lower levels of proteinuria compared to males, a difference which was abolished by castration but not by ovariectomy. Similarly, albuminuria was higher in males than in females, and interestingly, it was the highest in males in the oldest group of animals. Recent studies show that differences in proteinuria between male and female rat might be structural in origin. This further supports using Wistar model for the study of sex-dependent differences in glomerular filtration [33]. This is entirely consistent with earlier studies which show negative effects of testosterone on kidney function and glomerular filtration rate and renal blood flow [34–36]. The presence of proteinuria is a powerful indicator of probability of renal kidney disease in human [37, 38].

Regression analysis of ATGR1 expression and ED1 showed that they were associated (a positive moderate correlation *r* = 0.4; *P* < 0.001). Greater inflammation at 6 months of age was associated with a higher ATGR1/AGTR2 ratio (*r* = 0.3; *P* < 0.05). However, under our conditions, we did not see a clear relationship between the capacity to synthesise sex steroids and angiotensin II receptor expression across the 18-month period. ATG1R expression was highest at 6 months of age in all groups, again consistent with the phase of greatest inflammation. We are unsure why the inflammatory processes occurred in early adulthood but were dampened with ageing. It is possible that greater inflammation in earlier life left behind damage and functional deficit in the ageing animals, beyond the phase when inflammation had subsided. AGTR2 protein expression appeared to be higher in the gonadectomised females and then decrease with age in that group. Although AGTR1 protein expression was associated with greater inflammation, under our experimental conditions, the expression of AngII receptor proteins cannot explain the observed differences between the sexes or effects of gonadectomy upon renal function and blood pressure. Analysing AngII receptors expression by Western blot is challenging, and we must acknowledge some uncertainty about the validity of our measurements. Commercially available antibodies are not very well optimised and vary from batch to batch. Moreover, the type 1 receptor is heavily glycosylated in vivo and as a result has multiple molecular weights that exceed the single purified peptide. This influences findings regarding AGTR1 and AGTR2 expression and makes comparison between findings from different laboratories difficult. In this experiment, although we saw a single band with the size corresponding to the molecular size of the protein and we additionally used BLAST tool (from NCBI webpage) in order to check the specificity of the peptide used to produce the antibody, we were unable to assess specificity by more robust methods.

The telemetry blood pressure data showed that females have lower resting SBP (Fig. 5a) and also DBP (Fig. 5b). The difference between healthy normotensive males and females SBP was 4% and DBP 8%, and it was very consistent. DBP was lower in the sham animals compared to the gonadectomised, and this suggests that female and male hormones may play some role in the regulation of the blood pressure. The tail-cuff method did not replicate the differences in the SBP or DBP at 12 months of age observed by telemetry but did show a clear reduction in the SBP and DBP and increase in the HR of castrated males at 18 months suggesting that the observed change is very pronounced at this time point (Fig. 6a, b, c). However there was a tendency for SBP to be higher in males vs. in females (Fig. 6a, *P* = 0.07). Additionally a comparison of SBP between 12 and 18 months in females (*T* test) showed a significant difference of 7 mmHg (*P* < 0.01). This could be due to the impact of age-related decline in ovarian hormone production [39]. The discrepancy between blood pressures measured by telemetry and using an indirect tail-cuff method is of some interest. As described previously [22], tail-cuff pressures are prone to artefacts that are associated with stress but may also differ from central readings that are not obtained from resistance vessels due to factors such as the length of the arterial tree, length of the systole [40, 41], arterial stiffness [42] and activity of the autonomic nervous system. We might suspect a very distinct attenuation the vagal component of baroreflex sensitivity at 18 months of age in the castrated group as this was observed in a short-term study of castrated sexually matured male rats [43].

It is well established that circulation is affected by activity of the autonomic nervous system and that sympatho-vagal balance plays an important role in maintaining pressure [44]. Information on the autonomic regulation can be obtained by indirect (spectral analysis of the heart rate and systolic blood pressure) or direct (telemetry nerve recordings) measurements of the sympathetic nerve activity [24]. Circulation is affected by a cardiac cycle, respiration and vasomotor activity, and spectral analysis considers heart rate or arterial pressure as a sum of oscillatory components defined by their frequency and amplitude. In general, the low spectra are related to sympathetic and high spectra to the parasympathetic activities [45, 46].

In our study, gonadectomy did not affect cardiac sympatho-vagal balance in 12-month-old animals. Although we did not see any differences in the cardiac spectra, we did observe changes in the SBP spectra. Males and gonadectomised females show reduced HF% BP power which might suggests a sex difference in the effects of the respiration on the blood pressure. This would be in agreement with our data showing that the respiratory rates were higher in females than in males (Table 3). It seems that the VLF% were higher in males than in females suggesting that in males, sympathetic vasomotor tone might play an important role in the regulation of blood pressure. The increase in vasodilatory response in males compared to females has been shown in humans [47].

Our data show that spontaneous baroreflex gain was relatively higher in males but not affected by gonadectomy. Some of the human studies also show that males have higher baroreflex gain than females [48]. A more in-depth analysis of the blood pressure and heart rate variability at different ages, as well as contribution of such factors as the sympatho-adrenal nervous system to the regulation of the blood pressure, is needed to answer many remaining questions regarding sex differences in terms of regulating blood pressure.

## Conclusions

We cannot specifically attribute the effects of gonadectomy in males and females to sex hormones as there were no hormone replacement experiments to demonstrate this specificity. Overall, the study showed that the age-related decline in renal function is likely to be largely driven by androgens as the protective effect of orchidectomy was greater than the deleterious effect of ovariectomy.

## Additional file

Additional file 1: Figure S1. Specificity of AGTR1 antibody assessed by blocking peptide.

## Abbreviations

AGTR1: Angiotensin receptor type 1
AGTR2: Angiotensin receptor type 1
DBP: Diastolic blood pressure
F-GONADX: Ovariectomised females
F-INTACT: Intact females
HR: Heart rate
M-GONADX: Castrated males
M-INTACT: Intact males
SBP: Systolic blood pressure

## References

[1] Parati G, Ochoa JE, Salvi P, Lombardi C, Bilo G (2013). Prognostic value of blood pressure variability and average blood pressure levels in patients with hypertension and diabetes. Diabetes Care.

[2] Elperin DT, Pelter MA, Deamer RL, Burchette RJ (2014). A large cohort study evaluating risk factors associated with uncontrolled hypertension. J Clin Hypertens (Greenwich).

[3] August P, Oparil S (1999). Hypertension in women. J Clin Endocrinol Metab.

[4] Khoury S, Yarows SA, O’Brien TK, Sowers JR (1992). Ambulatory blood pressure monitoring in a nonacademic setting. Effects of age and sex. Am J Hypertens.

[5] Kearney PM, Whelton M, Reynolds K, Muntner P, Whelton PK, He J (2005). Global burden of hypertension: analysis of worldwide data. Lancet.

[6] Schenck-Gustafsson K (1996). Risk factors for cardiovascular disease in women: assessment and management. Eur Heart J.

[7] Losito A, Pittavini L, Ferri C, De Angelis L (2011). Kidney function and mortality in different cardiovascular diseases: relationship with age, sex, diabetes and hypertension. J Nephrol.

[8] Maas AH, Franke HR (2009). Women’s health in menopause with a focus on hypertension. Neth Heart J.

[9] Zanchetti A, Facchetti R, Cesana GC, Modena MG, Pirrelli A, Sega R, participants S (2005). Menopause-related blood pressure increase and its relationship to age and body mass index: the SIMONA epidemiological study. J Hypertens.

[10] Coylewright M, Reckelhoff JF, Ouyang P (2008). Menopause and hypertension: an age-old debate. Hypertension.

[11] Burt VL, Whelton P, Roccella EJ, Brown C, Cutler JA, Higgins M, Horan MJ, Labarthe D (1995). Prevalence of hypertension in the US adult population. Results from the Third National Health and Nutrition Examination Survey, 1988–1991. Hypertension.

[12] Macova M, Armando I, Zhou J, Baiardi G, Tyurmin D, Larrayoz-Roldan IM, Saavedra JM (2008). Estrogen reduces aldosterone, upregulates adrenal angiotensin II AT2 receptors and normalizes adrenomedullary Fra-2 in ovariectomized rats. Neuroendocrinology.

[13] Rogers JL, Mitchell AR, Maric C, Sandberg K, Myers A, Mulroney SE (2007). Effect of sex hormones on renal estrogen and angiotensin type 1 receptors in female and male rats. Am J Physiol Regul Integr Comp Physiol.

[14] McMullen S, Langley-Evans SC (2005). Maternal low-protein diet in rat pregnancy programs blood pressure through sex-specific mechanisms. Am J Physiol Regul Integr Comp Physiol.

[15] McMullen S, Langley-Evans SC (2005). Sex-specific effects of prenatal low-protein and carbenoxolone exposure on renal angiotensin receptor expression in rats. Hypertension.

[16] van Thiel BS, van der Pluijm I, te Riet L, Essers J, Danser AH (2015). The renin-angiotensin system and its involvement in vascular disease. Eur J Pharmacol.

[17] Silva-Antonialli MM, Tostes RC, Fernandes L, Fior-Chadi DR, Akamine EH, Carvalho MH, Fortes ZB, Nigro D (2004). A lower ratio of AT1/AT2 receptors of angiotensin II is found in female than in male spontaneously hypertensive rats. Cardiovasc Res.

[18] Baiardi G, Macova M, Armando I, Ando H, Tyurmin D, Saavedra JM (2005). Estrogen upregulates renal angiotensin II AT1 and AT2 receptors in the rat. Regul Pept.

[19] Baylis C (1994). Age-dependent glomerular damage in the rat. Dissociation between glomerular injury and both glomerular hypertension and hypertrophy. Male gender as a primary risk factor. J Clin Invest.

[20] Pijacka W, Clifford B, Tilburgs C, Joles JA, Langley-Evans S, McMullen S. Protective role of female gender in programmed accelerated renal aging in the rat. Physiol Rep. 2015;3:1–13. 10.14814/phy2.12342 PMC4425955 25902787

[21] Chung O, Kuhl H, Stoll M, Unger T (1998). Physiological and pharmacological implications of AT1 versus AT2 receptors. Kidney Int Suppl.

[22] Swali A, McMullen S, Langley-Evans SC (2010). Prenatal protein restriction leads to a disparity between aortic and peripheral blood pressure in Wistar male offspring. J Physiol.

[23] Cornock R, Langley-Evans SC, Mobasheri A, McMullen S (2010). The impact of maternal protein restriction during rat pregnancy upon renal expression of angiotensin receptors and vasopressin-related aquaporins. Reprod Biol Endocrinol.

[24] McBryde FD, Abdala AP, Hendy EB, Pijacka W, Marvar P, Moraes DJ, Sobotka PA, Paton JF (2013). The carotid body as a putative therapeutic target for the treatment of neurogenic hypertension. Nat Commun.

[25] Attia DM, Ni ZN, Boer P, Attia MA, Goldschmeding R, Koomans HA, Vaziri ND, Joles JA (2002). Proteinuria is preceded by decreased nitric oxide synthesis and prevented by a NO donor in cholesterol-fed rats. Kidney Int.

[26] Bongartz LG, Joles JA, Verhaar MC, Cramer MJ, Goldschmeding R, Tilburgs C, Gaillard CA, Doevendans PA, Braam B (2012). Subtotal nephrectomy plus coronary ligation leads to more pronounced damage in both organs than either nephrectomy or coronary ligation. Am J Physiol Heart Circ Physiol.

[27] Neuhaus OW, Flory W (1978). Age-dependent changes in the excretion of urinary proteins by the rat. Nephron.

[28] Roy AK, Neuhaus OW (1967). Androgenic control of a sex-dependent protein in the rat. Nature.

[29] Glucksmann A (1974). Sexual dimorphism in mammals. Biol Rev Camb Philos Soc.

[30] Dewan ZF, Morris ID, Lendon RG (2000). Administration of exogenous testosterone in the adult rat and its effects on reproductive organs, sex hormones and body-weight. Bangladesh Med Res Counc Bull.

[31] Saruhan BG, Ozdemir N (2005). Effect of ovariectomy and of estrogen treatment on the adrenal gland and body weight in rats. Saudi Med J.

[32] Shortliffe LM, Ye Y, Behr B, Wang B. Testosterone changes bladder and kidney structure in juvenile male rats. J Urol. 2014;191:1913–9. 10.1016/j.juro.2014.01.012 24518779

[33] Imafidon EC, Akomolafe RO, Oladele AA. Sexually dimorphic proteinuria in Wistar rats: relevance to clinical models. Pathophysiology. 2016;23:51–9. 10.1016/j.pathophys.2016.02.001 26896858

[34] Gafter U, Ben-Bassat M, Levi J (1990). Castration inhibits glomerular hypertrophy and proteinuria in uninephrectomized male rats. Eur J Clin Invest.

[35] Fortepiani LA, Yanes L, Zhang H, Racusen LC, Reckelhoff JF (2003). Role of androgens in mediating renal injury in aging SHR. Hypertension.

[36] Reckelhoff JF, Zhang H, Granger JP (1998). Testosterone exacerbates hypertension and reduces pressure-natriuresis in male spontaneously hypertensive rats. Hypertension.

[37] Gorriz JL, Martinez-Castelao A (2012). Proteinuria: detection and role in native renal disease progression. Transplant Rev (Orlando).

[38] Eddy AA (2004). Proteinuria and interstitial injury. Nephrol Dial Transplant.

[39] Hinojosa-Laborde C, Craig T, Zheng W, Ji H, Haywood JR, Sandberg K (2004). Ovariectomy augments hypertension in aging female Dahl salt-sensitive rats. Hypertension.

[40] Bortolotto LA, Safar ME (2006). Blood pressure profile along the arterial tree and genetics of hypertension. Arq Bras Cardiol.

[41] Nichols WWORM (1998). McDonald’s blood flow in arteries: theoretical, experimental and clinical principles.

[42] Safar ME, Blacher J, Mourad JJ, London GM (2000). Stiffness of carotid artery wall material and blood pressure in humans: application to antihypertensive therapy and stroke prevention. Stroke.

[43] Ward GR, Abdel-Rahman AA (2005). Effect of testosterone replacement or duration of castration on baroreflex bradycardia in conscious rats. BMC Pharmacol.

[44] Joyner MJ, Charkoudian N, Wallin BG (2010). Sympathetic nervous system and blood pressure in humans: individualized patterns of regulation and their implications. Hypertension.

[45] Malliani A, Pagani M, Lombardi F, Cerutti S (1991). Cardiovascular neural regulation explored in the frequency domain. Circulation.

[46] Malliani A, Pagani M, Lombardi F, Furlan R, Guzzetti S, Cerutti S (1991). Spectral analysis to assess increased sympathetic tone in arterial hypertension. Hypertension.

[47] Hart EC, Wallin BG, Barnes JN, Joyner MJ, Charkoudian N (2014). Sympathetic nerve activity and peripheral vasodilator capacity in young and older men. Am J Physiol Heart Circ Physiol.

[48] Barantke M, Krauss T, Ortak J, Lieb W, Reppel M, Burgdorf C, Pramstaller PP, Schunkert H, Bonnemeier H (2008). Effects of gender and aging on differential autonomic responses to orthostatic maneuvers. J Cardiovasc Electrophysiol.

