# Supplementary figures and images for: Impact of gonadectomy on blood pressure regulation in ageing male and female rats
Source: Biol Sex Differ. 2016 Dec 3;7:64. doi: 10.1186/s13293-016-0111-9 (PMC5135757; doi:10.1186/s13293-016-0111-9)

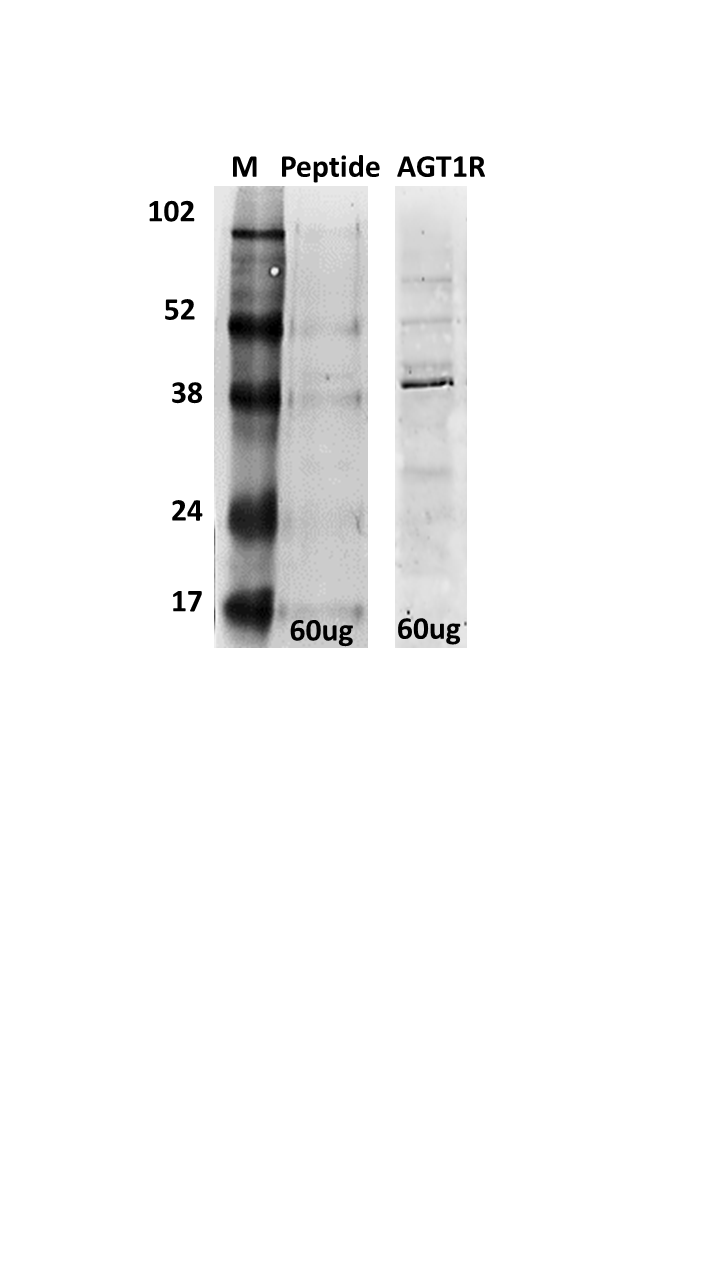

Supplement: Additional file 1: Figure S1. — Specificity of AGTR1 antibody assessed by blocking peptide. [file 13293_2016_111_MOESM1_ESM.tif]
